# Association between Combined Metals and PFAS Exposure with Dietary Patterns: A Preliminary Study

**DOI:** 10.3390/environments11060127

**Published:** 2024-06-18

**Authors:** Augustina Odediran, Emmanuel Obeng-Gyasi

**Affiliations:** 1 Department of Built Environment, North Carolina A&T State University, Greensboro, NC 27411, USA; 2 Environmental Health and Disease Laboratory, North Carolina A&T State University, Greensboro, NC 27411, USA

**Keywords:** chronic diseases, Dietary Inflammatory Index (DII), PFAS, heavy metals, NHANES, Bayesian kernel machine regression (BKMR), exposure–response relationships, cadmium, mercury, public health policies

## Abstract

**Background:**

The global burden of chronic diseases has been increasing, with evidence suggesting that diet and exposure to environmental pollutants, such as per- and polyfluoroalkyl substances (PFAS) and heavy metals, may contribute to their development. The Dietary Inflammatory Index (DII) assesses the inflammatory potential of an individual’s diet. However, the complex interplay between PFAS, heavy metals, and DII remains largely unexplored.

**Objective:**

The goal of this cross-sectional study was to investigate the associations between diet operationalized as the DII with individual and combined lead, cadmium, mercury, perfluorooctanoic acid (PFOA), and perfluorooctanesulfonic acid (PFOS) exposures using data from the National Health and Nutrition Examination Survey (NHANES) 2017–2018.

**Methods:**

Descriptive statistics, a correlational analysis, and linear regression were initially used to assess the relationship between the variables of interest. We subsequently employed Bayesian kernel Machine regression (BKMR) to analyze the data to assess the non-linear, non-additive, exposure–response relationships and interactions between PFAS and metals with the DII.

**Results:**

The multi-variable linear regression revealed significant associations between the DII and cadmium and mercury. Our BKMR analysis revealed a complex relationship between PFAS, metal exposures, and the DII. In our univariate exposure–response function plot, cadmium and mercury exhibited a positive and negative linear relationship, respectively, which indicated a positive and negative relationship across the spectrum of exposures with the DII. In addition, the bivariate exposure–response function between two exposures in a mixture revealed that cadmium had a robust positive relationship with the DII for different quantiles of lead, mercury, PFOA, and PFOS, indicating that increasing levels of cadmium are associated with the DII. Mercury’s bivariate plot demonstrated a negative relationship across all quantiles for all pollutants. Furthermore, the posterior inclusion probability (PIP) results highlighted the consistent importance of cadmium and mercury with the inflammatory potential of an individual’s diet, operationalized as the DII in our study, with both showing a PIP of 1.000. This was followed by PFOS with a PIP of 0.8524, PFOA at 0.5924, and lead, which had the lowest impact among the five environmental pollutants, with a PIP of 0.5596.

**Conclusion:**

Our study suggests that exposures to environmental metals and PFAS, particularly mercury and cadmium, are associated with DII. These findings also provide evidence of the intricate relationships between PFAS, heavy metals, and the DII. The findings underscore the importance of considering the cumulative effects of multi-pollutant exposures. Future research should focus on elucidating the mechanistic pathways and dose–response relationships underlying these associations in a study that examines causality, which will enable a deeper understanding of the dietary risks associated with environmental pollutants.

## 1. Introduction

The global burden of chronic diseases, including cardiovascular disorders, diabetes, and certain cancers, has been on the rise in recent decades [1,2]. Emerging evidence suggests that chronic inflammation and exposure to environmental pollutants, such as PFAS and heavy metals, may play a significant role in the development and progression of these diseases [3].

The intricate interplay between diet and environmental pollutants remains a critical yet underexplored domain within public health research [4]. This study sought to unravel the multi-faceted interactions between diet, characterized by the Dietary Inflammatory Index (DII), and cumulative exposures to per- and polyfluoroalkyl substances (PFAS) and heavy metals such as lead, cadmium, and mercury. Given the pervasive exposure to these complex contaminant mixtures, our study aimed to explore the nuanced associations between dietary patterns—specifically, those with pro- or anti-inflammatory potential—and the burden of multi-pollutant exposures.

The DII Is a novel tool designed to evaluate an individual’s diet’s inflammatory potential by analyzing the intake of various nutrients [5]. It is based on scientific evidence linking various dietary components with inflammation [6]. The DII has been validated through its correlation with several inflammatory markers and linked to multiple chronic diseases [7,8].

Several studies have underscored the integral role of inflammation in the pathogenesis of a broad spectrum of diseases, including cardiovascular diseases [9,10], diabetes [11], and cancer [12].

PFAS are a group of synthetic chemicals that are widely used in various industrial and consumer products, such as non-stick cookware, food packaging, and water-repellent fabrics [13]. These chemicals are persistent in the environment and have been detected in human blood and tissue samples worldwide [14,15]. Exposure to PFAS has been associated with various adverse health outcomes, including immune system dysfunction, endocrine disruption, and certain cancers [14,16-19].

Similarly, heavy metals, such as lead, cadmium, and mercury, are ubiquitous environmental pollutants that can accumulate in the human body over time. Chronic exposure to heavy metals has been linked to oxidative stress and systemic inflammation, potentially contributing to the etiology of chronic diseases such as neurodevelopmental disorders, kidney damage, and cardiovascular diseases [20-22].

Recent studies have suggested that combined exposure to PFAS and heavy metals may have synergistic effects on chronic diseases [23,24]. Additionally, dietary factors, such as the consumption of proinflammatory foods, may modulate or mediate the relationship between environmental pollutants and health outcomes [25-27].

The biological plausibility of these associations is supported by the known mechanisms of metal-induced toxicity and the ability of these pollutants to induce oxidative stress and disrupt immune function [28,29]. However, the complexity of exposure and response relationships, particularly for mixtures of metals and PFAS, has not been fully elucidated. This study explores the first crucial yet complex step in this relationship by examining the impacts of PFAS and metals on a diet.

To address this complexity, advanced statistical methods such as Bayesian kernel machine regression (BKMR), which have gained prominence in environmental health research [30], can serve as critical tools. BKMR allows for evaluating non-linear and non-additive relationships and potential synergistic interactions among exposures, providing a more comprehensive understanding of the effects of multi-pollutant exposures [30-32].

By leveraging the robustness of BKMR and focusing on the collective influence of the DII, PFAS, and heavy metals on diet, this study aims to advance our understanding of the intricate interplay between diet and environmental exposures [33]. The insights gained from this research will inform future studies and guide public health strategies to mitigate these exposures’ adverse effects and promote optimal health outcomes.

## 2. Materials and Methods

### 2.1. Study Cohort and Design

The National Health and Nutrition Examination Survey (NHANES) is a comprehensive, nationally representative dataset that provides valuable insights into the health and nutritional status of the non-institutionalized population in the United States. The NHANES dataset is collected by the U.S. Centers for Disease Control and Prevention (CDC) and released in two-year cycles. It employs a complex, multi-stage, stratified, and clustered sampling design.

This study used data from the NHANES 2017–2018 cycle. The NHANES dataset represents non-institutionalized individuals residing in all 50 U.S. states and the District of Columbia. The data collection process involves a physical examination and an interview for the selected participants. During the examination, blood samples are collected and sent to a laboratory for an analysis [34]. Detailed information regarding the study design, data collection procedures, and methodologies is available on the CDC’s NHANES website.

### 2.2. Calculation of DII Scores

In this study, we utilized data collected through 24 h dietary recall interviews, which had been previously validated by the Nutrition Methodology Working Group. The DII was calculated individually for each 24 h recall and the average DII value was used for our analysis.

Using Hébert’s scheme, the DII was computed [5,7]. The DII assesses dietary inflammation by evaluating the inflammatory impacts of 45 different food components, and is widely utilized for this purpose. The NHANES dataset encompassed 27 food parameters used to calculate the DII, including energy, carbohydrates, protein, and fiber. It also covered various fats such as total saturated fatty acids, total monounsaturated fatty acids, and polyunsaturated fatty acids (PUFA). Additionally, it included cholesterol, niacin, vitamins (B6, B12, A, C, D, E), minerals (magnesium, selenium, zinc, iron), and other components, such as thiamine, riboflavin, beta-carotene, folic acid, n-3 PUFA, n-6 PUFA, and alcohol. Using fewer food parameters can provide reliable predictions, according to prior studies [8,25].

### 2.3. Statistical Analysis

Before conducting the primary analyses, we performed data pre-processing to handle missing values and ensure data quality. Missing values within variables of interest were imputed using the median value to maintain the integrity of the dataset. A thorough data cleaning process was also undertaken to identify and resolve any inconsistencies, duplicate records, or irrelevant information. This preprocessing step was crucial in preparing a complete and reliable dataset for subsequent analyses, minimizing the potential for bias arising from missing or erroneous data.

The main analytical approaches employed in this study were linear regression and Bayesian kernel machine regression (BKMR). Linear regression was used to examine the associations between the predictor variables and the outcome of interest, while BKMR was applied to capture potential non-linear relationships and interactions among the predictor variables. By utilizing these complementary analytical techniques, we aimed to provide a comprehensive understanding of the relationships within our data.

#### 2.3.1. Descriptive Statistics

The descriptive statistics were calculated to describe the distributions of PFAS, metals, and demographic variables and to stratify them using the DII. Spearman correlations were used to assess the relationships among the study variables. To evaluate differences between participants, the survey-weighted *t*-test was used to compare means.

#### 2.3.2. Bayesian Kernel Machine Regression

Bayesian kernel machine regression (BKMR), a powerful analytical tool for investigating the combined effects of multiple pollutants on health outcomes, was used in this study. BKMR effectively captures complex interactions and dependencies within data, providing a more accurate assessment of the joint impact of contaminants on a specific outcome of interest [30,31]. In this study, we employed BKMR to evaluate the collective effect of PFOA, PFOS, mercury, lead, and cadmium on the DII.

We employed BKMR with the Markov chain Monte Carlo (MCMC) sampling method, adapted from the methodology outlined by Bobb et al. [32]. The analysis involved 5000 iterations. Posterior inclusion probabilities (PIPs), ranging from 0 to 1, were used to assess the impacts of individual metals and PFAS in the environmental mixture, quantifying their relative importance.

To understand the interactions between these metals and the outcome of interest, we computed high-dimensional exposure–response functions, h(z), at various intervals, while keeping other influencing variables constant at their median values. The graphical interpretation of the BKMR results allowed for a comparative analysis of each metal exposure’s collective and individual effects, contrasting outcomes at specific exposure percentiles against those at median exposure levels.

The model employed in this study was as follows:

$$g(\mu_{i})=h\;(z_{i}1,\ldots ,z_{i}M)+\beta X_{i};i=1,\ldots ,n$$

where g represents a monotonic link function; $\mu_{i}=E(Y_{i})$ is the health end point; h represents the flexible kernel function of exposures $z_{i}1,\ldots ,z_{i}M$; x is the vector of covariates for the i-th observation, including BMI, gender, age, ethnicity, alcohol consumption, smoking, and income; and β represents a vector of associated coefficients [1]. The analyses were adjusted for BMI, gender, age, ethnicity, alcohol consumption, smoking, and income. The analyses were completed using R (version 4.2.3; R Foundation for Statistical Computing, Vienna, Austria) [5], with the significance level set at 0.05.

## 3. Results

### 3.1. Characteristics of the Sample Population

Table 1 summarizes the age, BMI, lead, cadmium, mercury, PFOA, PFOS, and DII data. The mean age of the participants was 34 years and the mean BMI was 26.57.

Table 2 explores the mean levels of contaminants of interest and the Dietary Inflammatory Index, with proinflammatory indicated by ‘1’ (DII > 0) and anti-inflammatory indicated by ‘0’ (DII < 0). The results reveal that participants on an anti-inflammatory diet had higher mean levels of PFOA, PFOS, and mercury. Conversely, those on a proinflammatory diet had higher mean levels of cadmium and lead. None of these differences reached statistical significance. Regarding the DII, there was a statistically significant age difference, with younger individuals being more likely to be on a proinflammatory diet.

Regarding sociodemographic factors, this study consisted of 49.2 percent males and 50.7 percent females. The sociodemographic factors further explored the proportion of participants whose diet was classified as an anti-inflammatory diet (DII = 0) or a proinflammatory diet (DII = 1). The critical results indicated that those with an anti-inflammatory diet were to a greater degree male (62% versus 38%). See Table A1 in the Appendix A for further sociodemographic and behavioral variables within the study.

### 3.2. Correlation between Environmental Contaminants Variables and the DII

**Figure 1.**
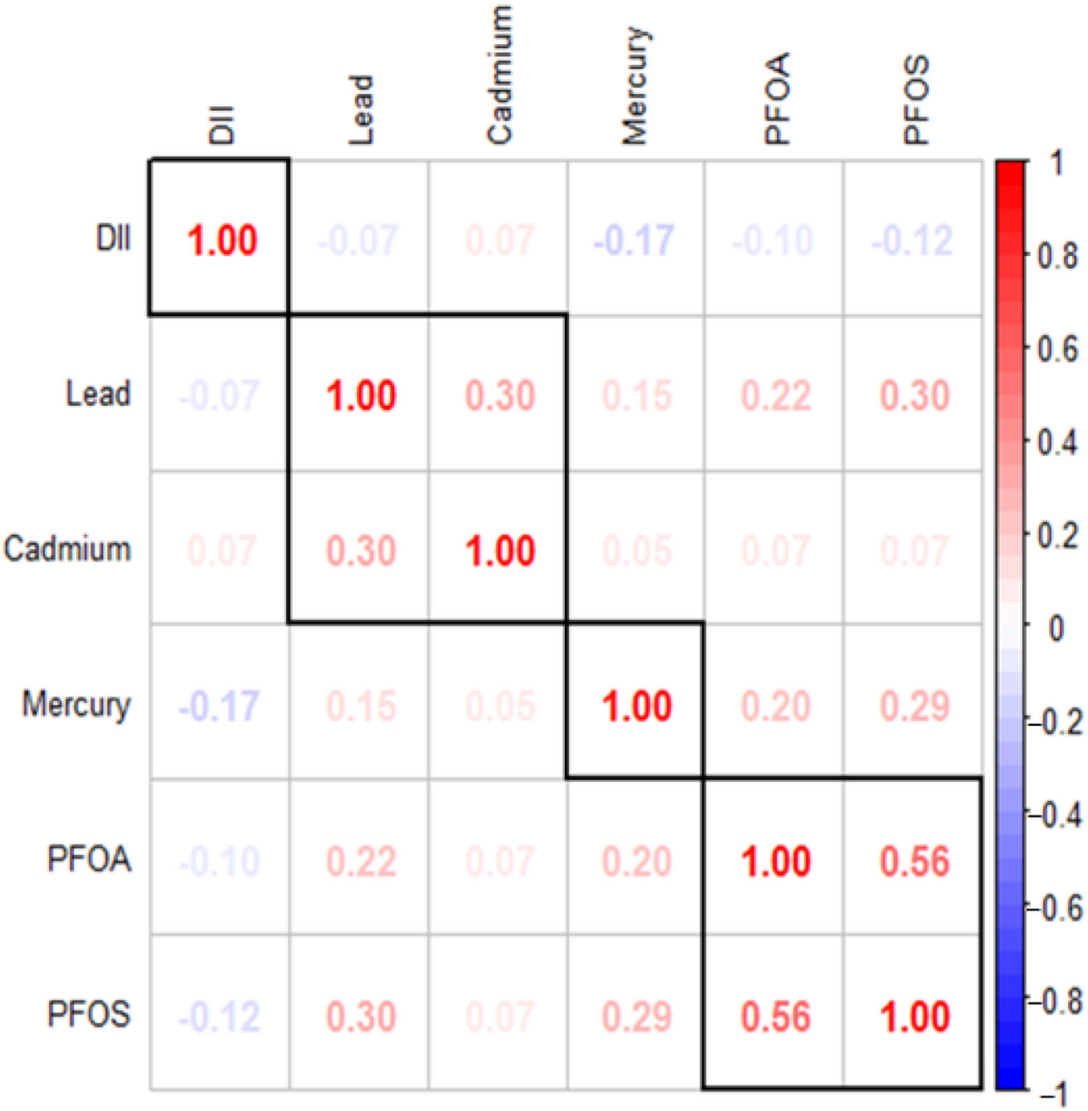
Spearman correlations among the DII, PFAS and metals. Dark red indicates strong positive correlation while dark blue indicates strong negative correlation.

Figure 1 illustrates the Spearman correlation matrix, which reveals the relationships between various environmental contaminants, including lead, cadmium, mercury, PFOA, PFOS, and the DII. The results indicate significant correlations among the DII, PFAS, and metals. Specifically, PFOS show a moderate positive correlation with PFOA (*ϱ* = 0.56) and weaker positive correlations with lead (ρ = 0.30) and mercury (*ϱ* = 0.29), suggesting potential co-occurrence or interactions among these pollutants. The statistical analysis revealed significant relationships (*p* < 0.05) among various variables. However, the correlations between lead and PFOA, mercury and PFOA, and lead and cadmium were relatively weak. In this context, correlations with *ϱ* values around 0.50–0.70 were considered moderate, while those below 0.30 were considered weak.

Table 3 shows the linear regression analysis, which assessed the relationships between various PFAS and metals and the Dietary Inflammatory Index (DII) after adjusting for critical covariates. Cadmium showed a significant positive association with the DII, indicating that higher cadmium levels are associated with increased DII scores. Mercury demonstrated a significant negative association with a decrease in DII score.

### 3.3. BKMR Analysis

Our study investigated complex relationships between the combined effect of PFAS and metals on the DII. Traditional linear regression methods, which assume straightforward relationships between variables, often fail to capture the nuanced dynamics of such interactions in real-world exposure data. In contrast, Bayesian kernel machine regression (BKMR) uses kernel functions and Bayesian inference to effectively identify non-linear and non-additive patterns and intricate interactions, surpassing linear models in depth and accuracy.

By applying BKMR, our analysis could unearth insights that linear approaches may not detect; specifically, BKMR can accommodate potential non-linearity and interactions between PFAS, metals, and the DII. This methodology enabled a more comprehensive exploration of the potential underlying mechanisms at play and facilitated the generation of novel hypotheses.

#### 3.3.1. Posterior Inclusion Probability of Environmental Contaminants with DII

Table 4 presents the posterior inclusion probability (PIP) scores for various environmental contaminants, including lead, cadmium, mercury, PFOA, and PFOS. The PIP score measures the probability of each contaminant’s role in explaining the variation observed in the DII scores. Notably, cadmium and mercury exhibited PIP scores of 1.000, indicating a dominant impact. These findings provided evidence for the inclusion of cadmium and mercury as important predictors in the study’s outcome. In contrast, lead, PFOA, and PFOS showed lower PIP scores of 0.5596, 0.5924, and 0.8524, respectively, indicating a lower inclusion probability than cadmium and mercury.

#### 3.3.2. Univariate Association of the DII and Combined PFAS and Heavy Metals

**Figure 2.**
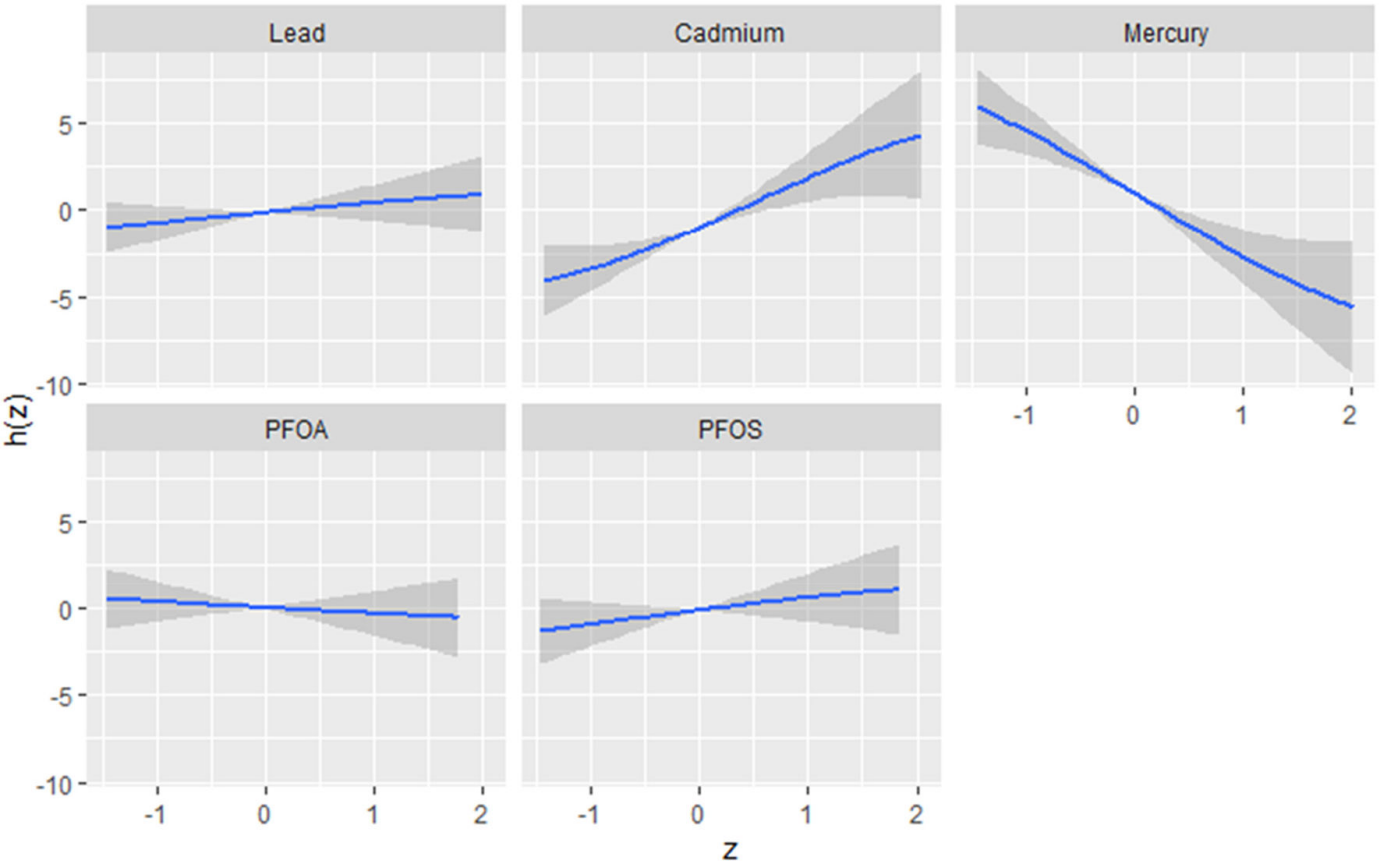
Univariate exposure–response function and 95% CI scores for the associations between single pollutant exposures when other pollutant exposures are fixed at the median level. Results adjusted for BMI, gender, age, ethnicity, alcohol consumption, smoking, and income.

The univariate approach was used to investigate the individualistic impacts of PFOA, PFOS, lead, cadmium, and mercury on the DII. Figure 2 displays the effects of individual PFAS and metals on the DII when other PFAS and metals are fixed at the median and the covariates are held constant. The results reveal cadmium and mercury have the greatest impact. The grey bands represent 95% confidence intervals. These functional forms, derived from the posterior distributions of the model parameters, provide valuable insights into the nature and complexity of the associations between the contaminants and the outcome. The plots reveal distinct patterns for each contaminant, highlighting the importance of considering their unique effects. Based on the figure, the flat curve in the lead panel suggests that variations in lead exposure do not significantly affect the DII scores across the range of exposure analyzed. This could mean that lead, within the study’s observed exposure range, might not impact the DII. In contrast, cadmium and mercury display strong associations with the DII, characterized by a steep increase. This indicates a profound positive effect, where the impact of these contaminants on the outcome remains robust at all exposure levels. Lead, PFOA, and PFOS show similar functional forms, displaying a flat relationship that indicates little to no effect on the DII.

#### 3.3.3. Bivariate Exposure–Response Function

**Figure 3.**
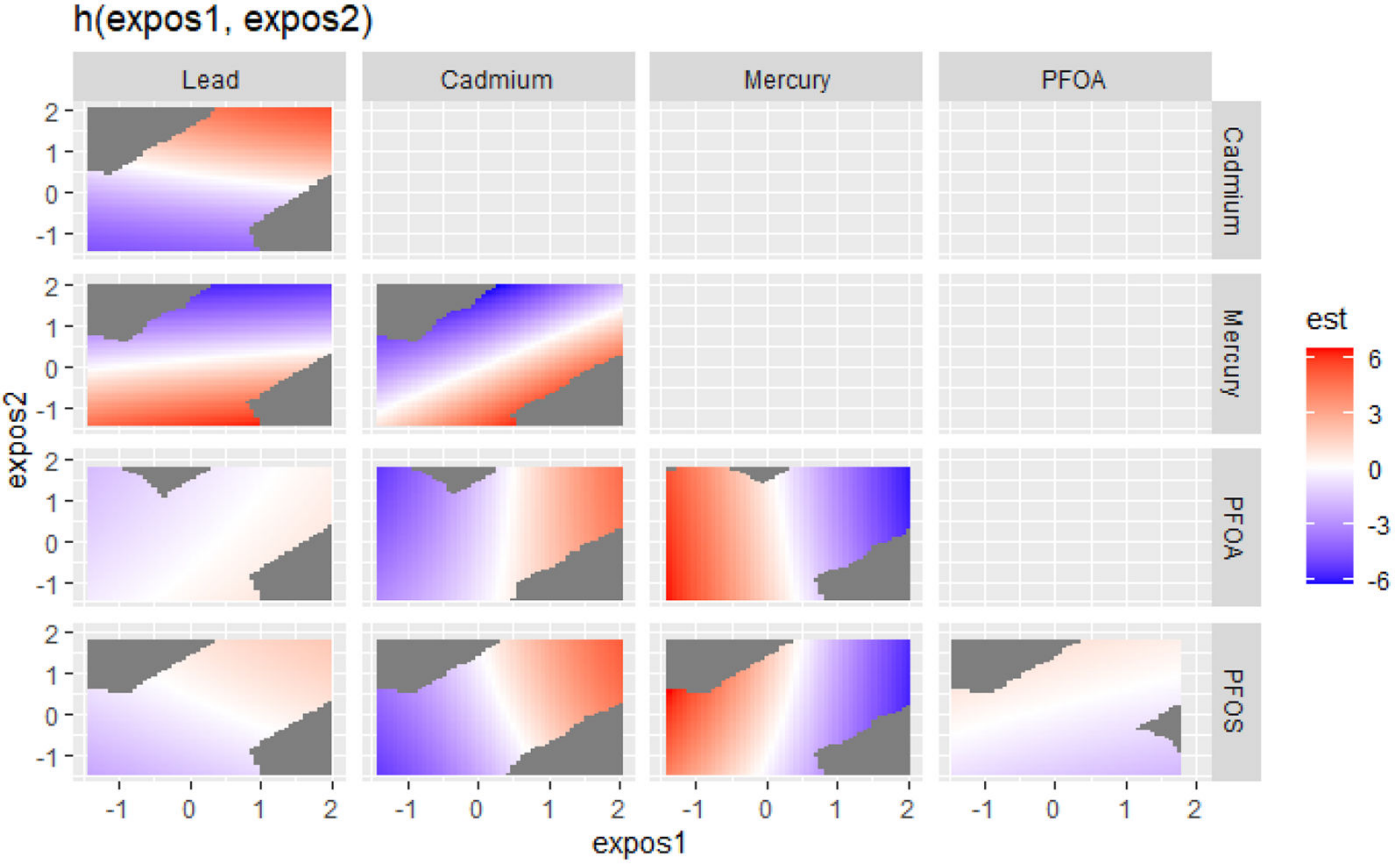
Bivariate exposure–response function of PFAS and metals with the DII. Results adjusted for BMI, gender, age, ethnicity, alcohol consumption, smoking, and income.

The effects of exposures to bivariate metals and PFAS on the DII were explored. Figure 3 visualizes the bivariate exposure–response functions for two predictors (expos1 and expos2) for lead, cadmium, mercury, PFOS, and PFOA, where all the other predictors are fixed at the 50th percentile and with adjustments for covariates. In the plot in Figure 3, the color scale (est) represents the estimated effects on health outcomes. Red indicates a higher positive effect (an increased risk of an adverse health outcome, associated with higher DII scores), blue indicates a negative impact, and white or grey indicates no effect. The plots reveal distinct patterns of association between expos1 and expos2 for the different contaminants. The results shown in Figure 3 suggest that in the lead vs. mercury (red region in the box), cadmium vs. mercury (red region in the box), mercury vs. PFOA, and mercury vs. PFOS plots, there appear to be regions where increasing levels of one contaminant are associated with a harmful effect on the outcome, as indicated by the red areas. This also happens with cadmium vs. PFOA and cadmium vs. PFOS, although to a lesser extent. The plots for lead vs. PFOA, lead vs. PFOS, and PFOS vs. PFOA show that higher levels of both exposures seem to have fewer effects on the DII, as indicated by the light red, white, and blue regions.

**Figure 4.**
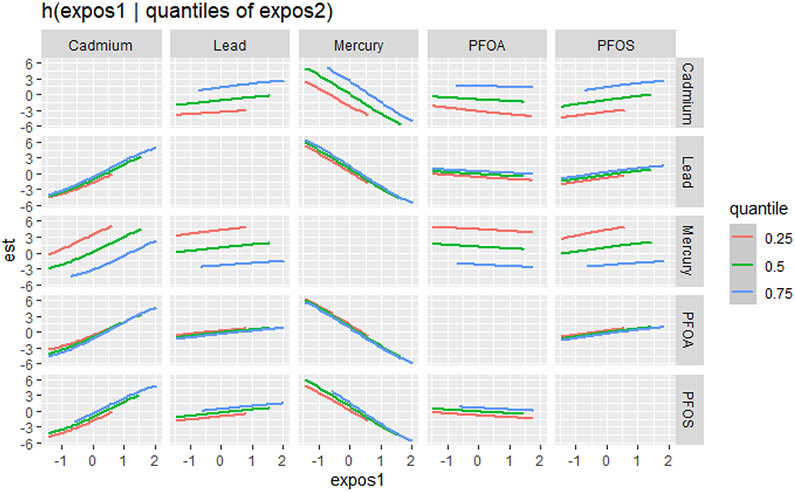
Bivariate exposure–response functions of PFAS and metals with the DII. This analysis investigated the predictor–response function with varying quantiles of the second predictor, while other predictors were fixed. Results adjusted for BMI, gender, age, ethnicity, alcohol consumption, smoking, and income.

The bivariate relationship was further explored by examining PFAS and metal pairs (Figure 4). The analysis assessed the relationships between individual metals and PFAS on the DII by fixing the other PFAS or metals at different quantiles (25th (red line), 50th (green line), and 75th (blue line)), with the remaining PFAS and metals held at the median level. These models were adjusted for the covariates of interest. The x-axis, labeled “expos1”, shows the levels of one exposure, while the y-axis, labeled “est”, represents the estimated effect on the DII. Each column of a plot corresponds to a different exposure, which is considered “expos1”.

Figure 4 presents the relationship between expos1 and the quantiles of expos2 for various environmental contaminants (cadmium, lead, mercury, PFOA, and PFOS). This innovative method allows for a more comprehensive understanding of the associations between the exposure variables across different quantiles of the response variable. The plots display the estimated quantile lines for each contaminant, with the color gradient representing different quantiles of expos2. The slopes of the lines indicate the strength and direction of the relationship between expos1 and expos2 at each quantile. The results reveal remarkable heterogeneity in the associations across contaminants and quantiles. Notably, the quantile lines are relatively parallel and positive for cadmium, suggesting a consistent positive relationship between this exposure (expos1) and other contaminants (expos2) across all quantiles. This indicates that increased levels of cadmium are consistently associated with higher DII scores, regardless of the exposure level. Mercury’s plots show a strong negative relationship with DII at different cadmium, lead, PFOA, and PFOS quantiles. These results suggest that mercury consistently exhibits a strong negative relationship with the DII when combined with other pollutants. In contrast, for PFOA, PFOS, and lead, the quantile lines are flat, suggesting that the relationship between these exposures (expos1) and other contaminants (expos2) is not significantly strong in any direction. These findings highlight the importance of considering the entire distribution of the response variable when studying the effects of environmental exposures.

#### 3.3.4. Overall Exposure Effect of the DII in Relation to PFAS and Heavy Metal Exposure Percentiles

**Figure 5.**
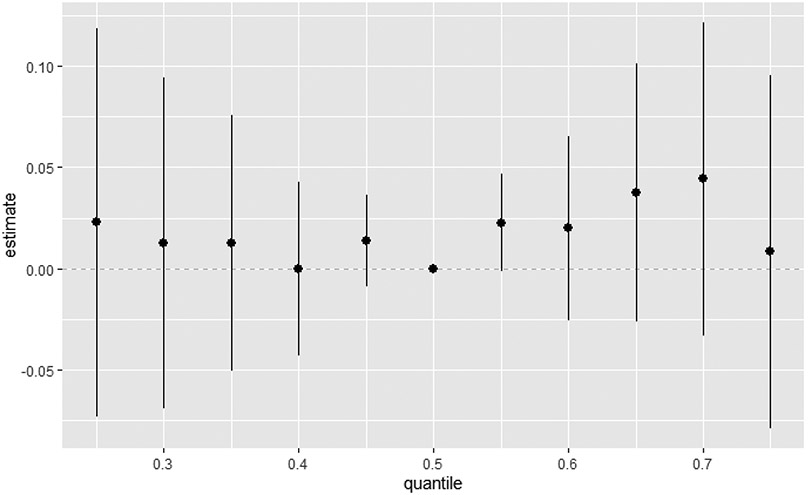
Summary of the overall health effects of the combined exposures on the DII at various quantiles (from 25th to 75th). Results adjusted for BMI, gender, age, ethnicity, alcohol consumption, smoking, and income.

Figure 5 explores the total effect of all environmental pollutants of interest together on the DII. This was explored across different quantiles from the 25th to the 75th quantile at an increment of 5 using the 50th percentile (median) to compare the exposures. The estimation for all exposures at the 50th percentile shown at zero (dashed line) demonstrates that when comparing all exposures between the 25th and 75th percentile exposure level to the 50th, the combined effect of PFAS and metals on the DII is positive, with the strongest signal being shown at the 70th percentile. Nevertheless, the confidence intervals suggest that the results should be interpreted with caution. The 50th percentile seems critical in defining how the combined effects of the pollutants manifest. This suggests a potential threshold effect, where the relationship between the quantile and the response variable changes abruptly once a certain quantile level is reached.

#### 3.3.5. Single-Variable Effects of PFAS and Metals with the DII

**Figure 6.**
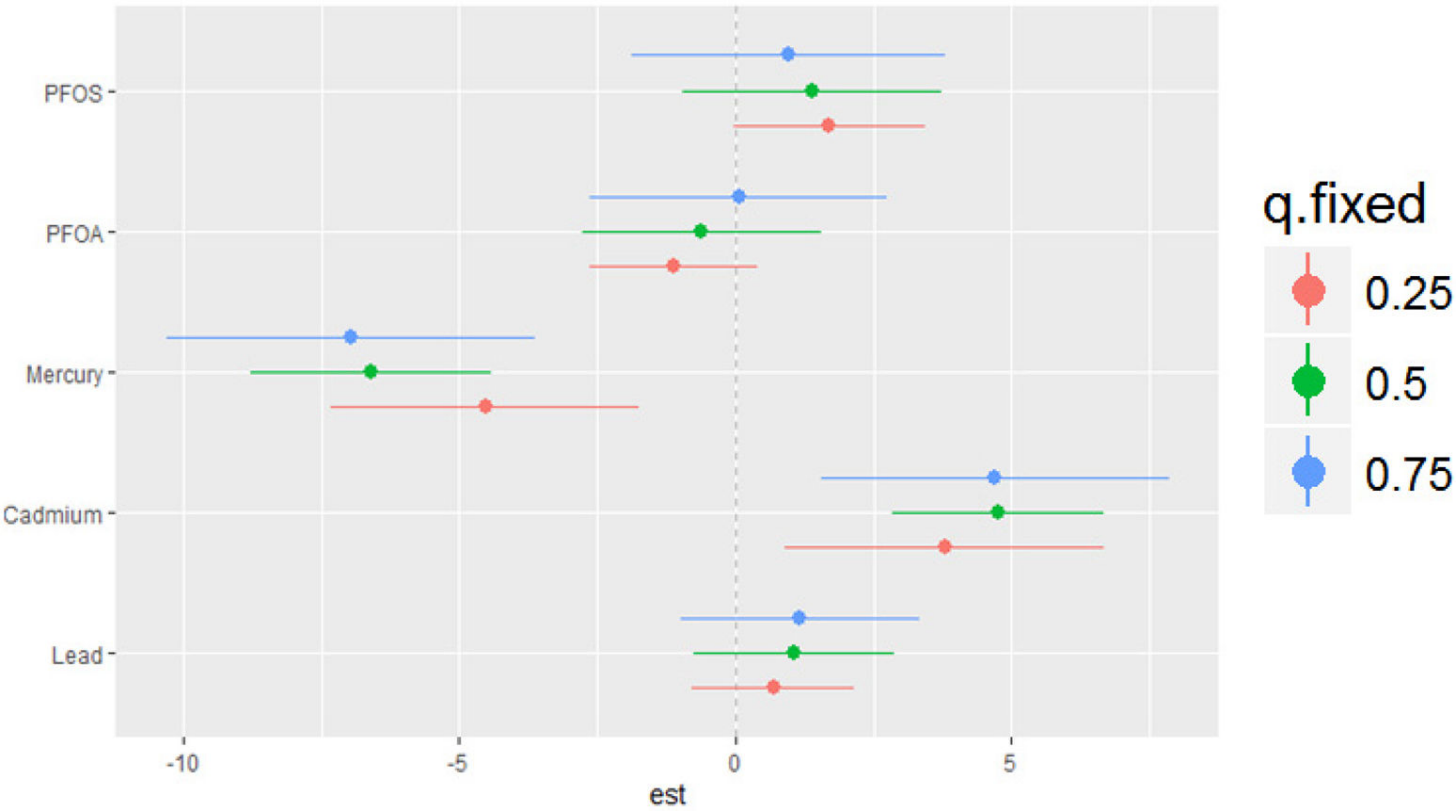
Single-variable effects of PFAS and heavy metals at increasing quartiles for the DII.

The single-variable effects helped to explore the impacts of a single predictor at different quantiles, allowing us to evaluate their contribution to the risk of higher DII scores. Figure 6 illustrates the single-variable effects of various PFAS and metals on the DII at the 25th, 50th, and 75th quantiles. PFOS, cadmium, and lead are positively related with DII at all three quantiles, indicating that higher levels of these pollutants are correlated with an increased risk of elevated DII. The effect appears to be more pronounced at the 75th percentile (blue line) compared to the 25th and 50th percentiles for lead, with wide variability among the other pollutants. The results indicate that higher cadmium levels are consistently associated with an increased risk of higher DII scores. The overall relationship of the pollutants within this study with the DII may also be non-additive and dependent on the level of exposure.

#### 3.3.6. Single-Variable Interaction Terms of PFAS and Metals on the DII

**Figure 7.**
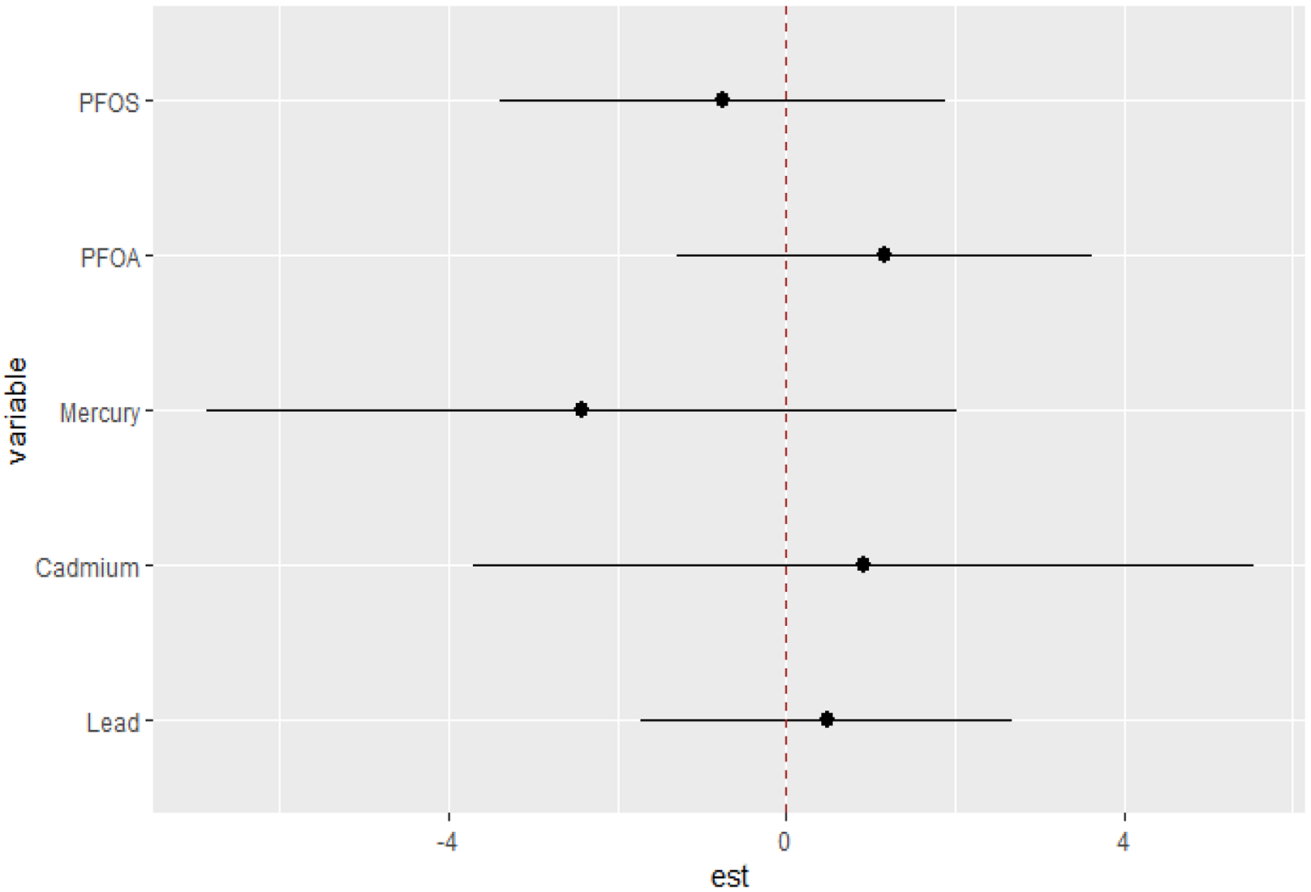
Single-variable interaction terms for PFOA, PFOS, mercury, cadmium, and lead at increasing quantiles for other PFAS and metals with the DII.

The potential interaction effects between PFAS and metals on the DII were explored. The analysis evaluated the likelihood of interaction inclusion for each variable pair, determining if their combined effect significantly enhanced the explanation of the DII outcome variable beyond their individual effects. Our findings as demonstrated in Figure 7 illustrate the overall impact of PFOA, PFOS, mercury, cadmium, and lead at higher quantiles. The charts compare the effects of a single variable when other PFAS and metal components are fixed at their 25th quantile versus their 75th quantile.

## 4. Discussion

This study involved a sophisticated analysis to unravel the intricate connections between PFAS, heavy metals, and the DII. Using BKMR, we revealed the subtle nuances that inform the associations between these environmental exposures and diet, as measured by the Dietary Inflammatory Index (DII).

The BKMR methodology allowed us to deeply explore the multi-faceted nature of our data, capturing the relationships and potential synergistic effects that often elude conventional linear regression models. This approach enabled us to comprehensively analyze how various heavy metals and PFAS, individually or cumulatively, contribute to DII scores.

Our findings reveal a complex interplay between PFAS, heavy metals, and the DII. The posterior inclusion probability scores highlight the dominant impacts of cadmium and mercury on the DII, suggesting their significant role in influencing dietary inflammation. This may be due to their overall contribution to inflammation [35], endocrine disruption [36,37], and oxidative stress [38]. The univariate exposure–response functions illustrate distinct patterns for each contaminant, with cadmium and mercury displaying strong non-linear associations with the DII. These results underscore the importance of considering the unique effects of individual pollutants when assessing their impacts on health outcomes.

The bivariate exposure–response functions further elucidate the potential synergistic effects between PFAS and metals. The observed relationships between lead and mercury, cadmium and mercury, mercury and PFOA, and mercury and PFOS suggest that the pollutants may amplify their impacts on the DII to varying extents, depending on the paired combination. These findings emphasize the need for a comprehensive approach to evaluating the health risks associated with multi-pollutant exposures [39,40].

Moreover, our analysis of the overall effect of exposure on the DII concerning PFAS and heavy metals revealed complex relationships. The marked shift observed at higher exposure percentiles suggests that the impacts of these pollutants on the DII may not be uniform across various levels of exposure. This finding underscores the importance of considering exposure levels when assessing the health risk and studying the underlying mechanisms driving this relationship. Investigating the factors contributing to the sudden increases at higher quantiles could provide valuable insights into the public health management of exposures and disease outcomes.

The single-variable effects of PFAS and metals on the DII provide further insights into their contributions to the overall risk of higher DII scores. The positive associations observed for PFOA, cadmium, and lead at different quantiles indicate that higher levels of these pollutants are consistently associated with an increased risk of dietary inflammation. Conversely, the negative association observed for mercury at higher quantiles speaks to the complexity of the relationship between mercury and the DII.

The differences between the linear regression results and the Bayesian kernel machine regression (BKMR) analysis highlight the complexity of environmental exposures and their interactions. However, both of these approaches identified mercury and cadmium as significant players in the relationship between PFAS and metals with diet. The potential antagonistic effects between mercury and other pollutants, as suggested by the bivariate analysis, indicate that mercury may have a dominant influence on the DII when combined with other contaminants. Overall, the ability of BKMR to capture potential interactions and account for confounders provided confirmation of and confidence in the linear regression results. Thus, the findings highlight the importance of using multiple analytical approaches to understand the full impacts of environmental exposures.

The implications of our findings extend beyond the realm of dietary inflammation. Given the well-established link between dietary inflammation and various chronic diseases, such as cardiovascular disorders, diabetes, and certain cancers [41], our results underscore the potential role of PFAS and heavy metal exposures in the potential development and progression of these conditions. In simple terms, a proinflammatory diet may be a critical mediator between environmental mixture exposure and disease outcome. By elucidating the complex interplay between these pollutants and the DII, our study provides valuable insights into the mechanisms through which environmental toxicants may contribute to the global burden of chronic diseases.

Furthermore, our findings highlight the importance of considering the cumulative effects of multi-pollutant exposures in public health policies and interventions [42,43]. The relationships observed between PFAS and metals suggest that addressing individual pollutants in isolation may not be sufficient to mitigate the health risks associated with these toxicants. Instead, a comprehensive approach that considers the complex mixture of environmental pollutants is warranted [42] before any mitigation approach is attempted, in case the pollutants produce non-additive and interactive relationships.

Our study also underscores the need for future research to further elucidate the mechanisms underlying the associations between PFAS, heavy metals, and the DII. While our analysis provides compelling evidence by using multiple analytical approches to confirm the relationships between multiple pollutants, additional studies are needed to unravel the biological mechanisms through which multi-pollutant exposures influence diet and chronic disease risk to ensure causality rather than associations are driving decision-making.

Moreover, our study’s potential threshold effects and non-linear relationships highlight the importance of considering exposure levels in future research and risk assessments. Further investigations into the dose–response relationships between PFAS, heavy metals, and the DII may provide valuable insights into the critical exposure levels at which these pollutants harm health.

Our study primarily investigated the associations between the Dietary Inflammatory Index (DII) and toxic exposures to metals and PFAS, and these relationships have several potential causes. Firstly, specific dietary habits might increase an individual’s exposure to certain pollutants, such as fish high in mercury and rice high in other metals, depending on the irrigation sources. Additionally, it is theoretically possible for toxic exposures to influence dietary habits; this scenario is less plausible for the pollutants we studied, although their known neurotoxicant effects suggest that this hypothesis requires further exploration. Thirdly, and most likely, a confounding factor such as socioeconomic status, lifestyle, housing conditions, or demographic characteristics might influence dietary habits and toxic exposures. While we adjusted for several potential confounders in our models, future research studies should focus on analyzing specific food items and other unmeasured factors to understand these complex relationships better.

### Limitations

One limitation of our study was the inability to explore the nuanced interactions of pollutant contamination with the diet fully; for example, future research studies should consider case studies involving populations with distinct dietary habits and exposure profiles, incorporate biomonitoring data for a more comprehensive understanding. In addition, while the DII provides a broad measure of the dietary inflammation potential, future studies could benefit from analyzing specific food items to better understand these direct relationships. Additionally, our study’s cross-sectional design limited our ability to infer the causality and temporality of the observed associations. The reliance on self-reported dietary intake data may also introduce recall and misclassification biases. Furthermore, the generalizability of our findings may be constrained by the specific population sample and geographic area we studied. Future longitudinal and mechanistic studies are needed to better establish the temporal relationships and causative pathways between environmental exposures and diet. Finally, the selection of the DII parameters was guided by the need to ensure that the parameters included were well-represented and accurately measured within the dataset. We utilized 27 food parameters to calculate the DII scores, based on their availability and quality within the data. The importance of polyphenols in dietary assessments is well-recognized due to their chelating capacities, especially in relation to metals such as cadmium. However, the primary limitation in this study was the NHANES dataset, which did not comprehensively capture detailed intake data for specific polyphenols. Consequently, polyphenols were not included in our DII calculations. This limitation highlights the need for more comprehensive dietary data collection in future studies to enable the inclusion of such important dietary components.

## 5. Conclusions

Our study comprehensively analyzed the complex interplay between PFAS, heavy metals, and the DII, revealing the intricate relationships and potential synergistic effects between these environmental toxicants and dietary inflammation. By employing advanced statistical methods, such as BKMR, we uncovered the subtle nuances and non-linear relationships that often elude traditional analytical approaches. Our findings highlight the critical need to consider the cumulative effects of multi-pollutant exposures in health outcomes and public health policies, moving beyond the current focus on single contaminants. Understanding these complex pathways and dose–response relationships is essential, as they pose significant health risks at individual and population levels.

## Figures and Tables

**Table 1.** Mean levels of critical variables of interest, including age in years, BMI, lead, cadmium, mercury, PFOA, PFOS and the Dietary inflammatory index.

Variable	* Participants (n)	Mean	Standard Error (SE)	Minimum	Maximum
Age (Years)	9254	34.3	25.5	0.00	80.0
BMI (kg/m^2^)	8005	26.6	8.26	12.3	86.2
Lead (μg/dL)	6884	1.08	1.29	0.050	42.5
Cadmium (μg/L)	7513	0.374	0.503	0.070	13.0
Mercury (μg/L)	7513	1.14	2.27	0.200	63.6
PFOA (ng/mL)	1929	1.71	1.82	0.140	52.9
PFOS (mg/mL)	1929	6.51	7.74	0.140	105
DII	7495	1.79	1.59	−4.34	5.15

* Participants indicates numbers of individuals who answered questions or provided data or specimens used for variables.

**Table 2.** Mean levels of critical variables of interest based on Dietary inflammatory index (DII) status.

	Mean	Std. Error	95% Confidence Interval	*p*-Value
PFOA				
0	1.92	0.154	1.59, 2.25	0.137
1	0.170	0.066	1.56, 1.84	
PFOS				
0	6.56	0.504	5.48, 7.63	0.067
1	5.64	0.219	5.18, 6.11	
Lead				
0	1.06	0.073	0.904, 1.22	0.263
1	1.00	0.054	0.957, 1.16	
Cadmium				
0	0.357	0.024	0.307, 0.409	0.360
1	0.382	0.014	0.352, 0.411	
Mercury				
0	1.51	0.079	1.19, 1.66	<0.0001
1	1.07	0.060	0.929, 1.17	
Age in Year				
0	46.0	0.875	44.1, 47.8	<0.0001
1	37.0	0.509	35.9, 38.1	
BMI				
0	28.0	0.351	27.2, 28.7	0.390
1	28.0	0.237	27.1, 28.1	

Estimations of the mean of each pollutant in relation to a proinflammatory diet, represented by 1, and an anti-inflammatory diet, represented by 0.

**Table 3.** Linear regression of associations between the DII and pollutants of interest.

DII	Coefficient *	Std. Error	*p*-Value	95% Confidence Interval
PFOA	−0.035	0.053	0.520	−0.149, 0.079
PFOS	−0.008	0.011	0.471	−0.030, 0.015
Lead	0.016	0.058	0.787	−0.108, 0.140
Cadmium	0.369	0.013	0.012	0.092, 0.647
Mercury	−0.123	0.031	0.001	−0.189, −0.057

* Adjusted for age, BMI, ethnicity, and alcohol consumption.

**Table 4.** PIP scores assessing the influence of lead, cadmium, mercury, PFOA, and PFOS on the DII.

Variable	PIP
Lead	0.560
Cadmium	1.000
Mercury	1.000
PFOA	0.592
PFOS	0.852

## Data Availability

The NHANES dataset is publicly available online, accessible at https://wwwn.cdc.gov/nchs/nhanes/ (accessed on 12 December 2023).

## Appendix A

**Table A1.** Proportion estimations of sociodemographic and behavioral variables based on the Dietary Inflammatory Index. Note: 1 = proinflammatory; 0 = anti-inflammatory.

	Proportion	Std. Error	95% Confidence Interval
Gender @ DII			
Male—0	0.624	0.031	0.556, 0.689
Male—1	0.453	0.010	0.432, 0.475
Female—0	0.375	0.031	0.311, 0.444
Female—1	0.547	0.010	0.525, 0.568
Ethnicity @DII			
1—0	0.101	0.020	0.0661,0.153
1—1	0.0875	0.016	0.0589, 0.128
2—0	0.0748	0.012	0.0533,0.104
2—1	0.0664	0.009	0.0492, 0.0889
3—0	0.628	0.034	0.553, 0.697
3—1	0.630	0.025	0.575, 0.682
4—0	0.0781	0.012	0.0555, 0.109
4—1	0.120	0.017	0.0875, 0.162
5—0	0.0655	0.011	0.0458, 0.0927
5—1	0.0504	0.009	0.319, 0.0721
6—0	0.0522	0.016	0.0266, 0.0997
6—1	0.0464	0.005	0.0367, 0.0585
Alcohol @ DII			
Yes—0	0.941	0.010	0.915, 0.960
Yes—1	0.914	0.006	0.898, 0.928
No—0	0.0588	0.010	0.0403, 0.0853
No—1	0.0858	0.007	0.0724, 0.102
Smoking @DII			
Yes—0	0.400	0.227	0.352, 0.449
Yes—1	0.419	0.0167	0.384, 0.455
No—0	0.600	0.023	0.551, 0.648
No—1	0.581	0.017	0.545, 0.616

Note: DII—Dietary Inflammatory Index: 1—Proinflammatory; 0—Anti-Inflammatory. Ethnicity: 1—Mexican American; 2—Other Hispanic; 3—Non-Hispanic White; 4—Non-Hispanic Black; 5—Non-Hispanic Asian; 6—Other Race, Including Multi-Racial.
